# Application of Cheese Whey Containing Postbiotics of *Lactobacillus acidophilus* LA5 and *Bifidobacterium animalis* BB12 as a Preserving Liquid in High-Moisture Mozzarella

**DOI:** 10.3390/foods11213387

**Published:** 2022-10-27

**Authors:** Houshmand Sharafi, Mehran Moradi, Saber Amiri

**Affiliations:** 1 Department of Food Hygiene and Quality Control, Faculty of Veterinary Medicine, Urmia University, Urmia 1177, Iran; 2 Department of Food Science and Technology, Faculty of Agriculture, Urmia University, Urmia 1177, Iran

**Keywords:** cheese whey, shelf life, probiotic bacteria, active packaging, cell free supernatant

## Abstract

High-moisture mozzarella cheese (HMMC) is a highly perishable cheese with a short shelf life. In this study, the effects of UF cheese whey containing postbiotics from *Lactobacillus acidophilus* LA-5 (P-LA-5), *Bifidobacterium animalis* BB-12 (P-BB-12), and their combination on the microbial (i.e., psychrophiles, mesophiles, lactic acid bacteria, and mold-yeast population) and sensory properties of HMMC were investigated. Postbiotics were prepared in a cheese whey model medium as a novel growth media and used as a preserving liquid in HMMC. The results demonstrate that postbiotics reduced the growth of all microorganisms (1.5–2 log CFU/g reduction) and P-LA5 and P-BB12 had the highest antibacterial performance on mesophiles and psychrophiles, respectively. Mold and yeast had the highest susceptibility to the postbiotics. Postbiotics showed a significant effect on maintaining the microbial quality of HMMC during storage, proposing postbiotics as a new preserving liquid for HMMC.

## 1. Introduction

Bioprotection and biopreservation of dairy products with microorganisms are innovative approaches to food safety and preservation [1,2]. Postbiotics are by-products of beneficial microorganisms, primarily lactic acid bacteria (LAB), which are produced in culture media, food, or the intestine. In spite of the absence of a clear and accepted definition [3,4,5], postbiotic constituents are various intracellular and extracellular compounds and it is broadly agreed that the removal of the bacterial cells is a requirement [6,7]. The postbiotic solution contains compounds with specific chemical structures that are safe for consumption and have a high shelf life for use in food [8]. The constituents include vitamins, organic acids such as lactic acid, short-chain fatty acids, proteins, secreted and bioactive peptides, bacteriocins, neurotransmitters, secreted biosurfactants, and bacterial enzymes. They play many well-known roles in food preservation and packaging such as controlling pathogens, destroying microbial biofilms, and biodegrading harmful chemical contaminants such as mycotoxins, pesticides, and other chemicals [9,10,11,12]. It has recently been demonstrated that bacterial postbiotics can be used as an antimicrobial compound in meat [13,14] sausage [15], chicken fillet [16] packaging, and preservation. Other potential applications of bacterial postbiotics as biopreservatives have also been investigated in various kinds of cheeses such as fresh cheese [17] and Prato cheese [18]. This evidence shows that bacterial postbiotics have broad-spectrum antimicrobial activity against both Gram-positive and Gram-negative bacteria and fungi [19].

High-moisture mozzarella cheese (HMMC) is a soft fresh-flavored Italian cheese that is produced by lactic acid bacteria (fermented mozzarella) or by the direct injection of organic acids (acidified mozzarella) in milk [20]. HMMC is cut into various forms, mainly in the form of balls or pearls, and generally stored in a liquid composed of tap water or diluted salt solution (NaCl or CaCl_2_) with a shelf life of 1–2 weeks depending on the packaging and storing conditions [21,22]. Due to the presence of a broad range of microorganisms and the mass exchange (i.e., migration of salt and water) between the product and the liquid preservative, which occurs during storage, it has a relatively short shelf life that is associated with the moisture content, production methods, and storage conditions [21,23]. Although HMMC is subjected to heat treatment, it may be contaminated post-process, resulting in cheese spoilage and serious health risks to consumers. The main microorganisms that can cause HMMC spoilage are psychrophilic bacteria, causing pigmentation, discoloration, and a bad taste by releasing protease and lipase enzymes [24]. Increasing the shelf life of HMMC due to its high popularity among consumers and increasing distribution is a major issue in the dairy industry. Multiple approaches have been designed to increase the shelf life including active packaging by coatings based on carboxymethyl cellulose and natamycin [25], active compounds such as potassium sorbate, sodium benzoate, calcium lactate, and calcium ascorbate [22], or adding bioactive compounds such as bergamot juice concentrate in mozzarella liquid [26].

*Lactobacillus acidophilus* LA5 and *Bifidobacterium animalis* BB12 are among the probiotics widely used in dairy products. In the commercial manufacturing of probiotics, postbiotics are generated and considered as a waste. Postbiotics contain different biological compounds such as antimicrobial peptides [27], organic acid [28], conjugated linoleic acid [29], and exopolysaccharides [30]. Then, the postbiotics solution, as a waste solution, can be applied as a low-cost and biologically active alternative source to the common preserving liquid for HMMC packaging in order to control cheese spoilage during storage and improve the shelf life. In almost all of the published literature on the use of LAB postbiotics in food, the authors used de Man Rogosa and Sharpe (MRS) to prepare postbiotic solutions. In this sense, it is particularly important to find new cheap and useless agro-industrial waste for the preparation of postbiotics. In this study, postbiotic solutions from *L. acidophilus* LA-5 (P-LA-5) and *B. animalis* BB-12 (P-BB-12) were prepared in a cheese whey model medium as a novel growth medium and used as a preserving liquid in HMMC in the form of active packaging. The effect of each postbiotic solution and their combination (P-LA-5 + BB-12) on the microbial, chemical, and sensory properties of HMMC was investigated.

## 2. Materials and Methods

### 2.1. Probiotic Strains and Cultures Preparation

Probiotic bacteria (*L. acidophilus* LA-5 and *B. animalis* BB-12) were obtained from Chr. Hansen (Hørsholm, Denmark). They were weighed separately according to the company’s recommendations and cultured in MRS broth (Merck, Darmstadt, Germany) containing 0.05 *w*/*v*% L-cysteine (Merck) and 0.1 *w*/*v*% lithium chloride (Merck) at 37 °C for 24 h. The bacterial cells were collected by centrifuging the culture media at 5000× *g* for 15 min. Finally, the pellets were washed twice with normal saline and suspended in peptone water (0.1%) (Merck, Darmstadt, Germany) to make a suspension containing 10^10^ CFU/mL [29].

### 2.2. Preparation of Postbiotics Solutions

Ultrafiltered (UF) cheese whey solution (total solid 5.6%), which is obtained during UF white cheese production, was provided by the Pegah dairy factory (Urmia, Iran) and used as a culture media for postbiotic preparation. Initially, the pH was adjusted to 4.5 with 5N hydrochloric acid (Merck, Darmstadt, Germany), then autoclaved at 115 °C for 15 min, and the precipitates were separated by centrifugation at 2360× *g* for 5 min. The pH of the media (50 mL) was adjusted to 4.5 and autoclaved at 121 °C for 15 min in 100 mL flasks. Yeast extract (1% *w*/*v*) (Quelab Laboratories, Quebec, QC, Canada) was then added using a syringe filter (pore size 0.45 μm) (Merck, Darmstadt, Germany). The method of Amiri et al. [27] was used for postbiotic preparation in UF cheese whey. Briefly, the whey media (100 mL) was inoculated with 1 mL of probiotic suspension (~10^10^ CFU/mL) and incubated at 37 °C for 36 h (bacterial growth was monitored visually and by optical density measurement at 12-h intervals), following which it was centrifuged at 7000× *g* for 30 min. The remaining bacterial cells were removed from the postbiotic solutions using a syringe filter (0.45 μm) [6] and the postbiotic solutions were used fresh for cheese preservation.

### 2.3. Cheese Application of Postbiotics

HMMC was freshly purchased from the Kaleh Company representative in Urmia, Iran with a 7-day refrigerator shelf life and used the day after production. Based on data provided by the company, HMMC was manufactured by direct acidification with citric acid. HMMC balls (25–30 g) were individually packaged in a specified volume (31.25 mL solution per each ball) of unconcentrated P-LA-5, P-BB-12, and P-LA-5 + BB-12 solutions (pH ≈ 3.8). The control samples were packed with sterile distilled water. All samples were stored at 7 °C for 16 days and the microbial characteristics, color, pH, and sensorial properties were analyzed every 4 days.

### 2.4. Cheese Analysis

#### 2.4.1. Microbiological Analysis

Five grams of HMMC was diluted in 45 mL of peptone water (0.1%) (Merck) and homogenized in a stomacher (Seward Medical Ltd., London, UK). The suspension was subsequently diluted and the appropriate dilutions were plated in the selective culture media for total psychrophilic count (TPC) [on Plate count agar (PCA; Ibresco, Tehran, Iran) supplemented with cycloheximide (100 mg/L) (Sigma Chemical, Saint Louis, MO, USA), and incubated at 7 °C for 10 days], total mesophilic count (TMC) [on PCA containing cycloheximide and incubated at 37 °C for 48 h], LAB bacilli count [on MRS agar containing cycloheximide and incubated under the anaerobic condition at 37 °C for 48 h], and yeast-mold count (YMC) [on yeast glucose chloramphenicol agar and incubated at 25 °C for 5 days] [21,22,31,32].

#### 2.4.2. pH Measurement

Five grams of HMMC with 45 mL of peptone water (0.1%) was homogenized by a stomacher and the pH was measured by a pH meter (Metrohm, Herisau, Switzerland).

#### 2.4.3. Sensorial Evaluation

Sensory evaluation was assessed using a 9-point hedonic scale (1 = dislike extremely; 2 = dislike very much; 3 = dislike moderately; 4 = dislike slightly; 5 = neither like nor dislike; 6 = like slightly; 7 = like moderately; 8 = like very much; 9 = like extremely) by 15 semi-trained assessors. The cheese was evaluated for odor, texture (elasticity (tactile), and overall acceptability (all attributes together) [21,22,32].

#### 2.4.4. Color Measurements

The color of the cheese samples was measured using a digital colorimeter (Lovibond, London, UK) on days 0, 4, 8, 12, and 16. Then, the total color difference (∆E) of each sample was obtained using the L*, a*, and b* values according to the following equation [33].

(1) $$\Delta E=\sqrt{{\left(\Delta L^{*}\right)}^{2}+{\left(\Delta a^{*}\right)}^{2}+{\left(\Delta b^{*}\right)}^{2}}$$

### 2.5. Statistical Analysis

Three biological replicates were performed for all treatments and each analysis was conducted in duplicate. The data obtained from this study were analyzed by analysis of variance (ANOVA (with GraphPad Prism version 5.0 for Windows (San Diego, CA, USA). Duncan’s multiple range test was performed to assess significant differences (*p* < 0.05) between the groups.

## 3. Results and Discussion

### 3.1. Microbiological Analysis

**Figure 1:**
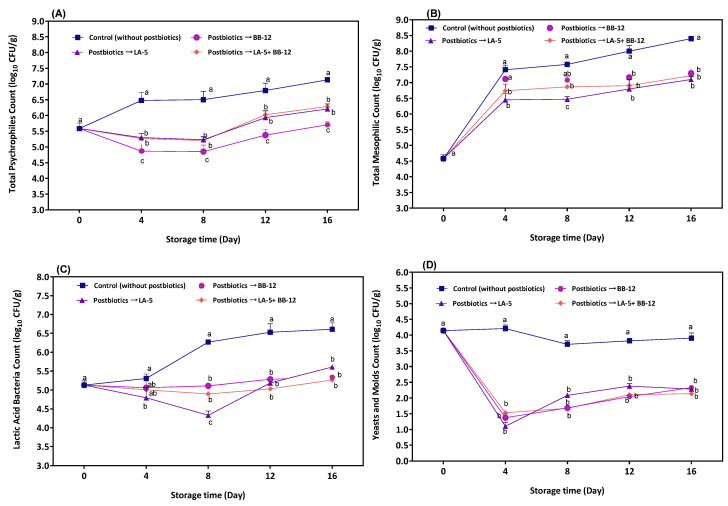
Changes in A: psychrophiles (**A**), mesophiles (**B**), lactic acid bacteria (**C**) and yeast and mold (**D**) population in mozzarella cheeses preserved in postbiotic solutions from *L. acidophilus* LA-5 (P-LA-5), *B. animalis* BB-12 (P-BB-12), and their combination during storage at 7 °C. Different lowercase letters indicate a significant (*p* < 0.05) difference between treatments on each storage day.

In the current work, the potential antimicrobial performance of the P-LA5, P-BB12, and their combination on TPC, TMC, LAB bacilli, and YMC of HMMC were evaluated during storage at 7 °C. The results demonstrated that P-LA5, P-BB12, and their combination had different inhibitory activity in all of the investigated microbial groups depending on the storage time, the type of microbial group, and postbiotic solutions (Figure 1). Active preserving liquid can be used to inhibit the growth of pathogens and spoilers and develop the physicochemical characteristics in HMMC [22,34]. For instance, the application of calcium lactate buffered brine as a preservative liquid provided enhanced protection against the microbial deterioration of HMMC (1–2 log CFU/g reduction in total viable count) [21]. Previous research has indicated that antimicrobial peptides [27,35], organic acids such as lactic acid [28], and bioactive lipids [29] are responsible for P-LA5 and P-BB12 antimicrobial activities. Moreover, P-LA5 contains possible antimicrobial compounds such as pyrrolo [1,2-a] pyrazine-1,4-dione, ethanol, threitol, phenol, cycloheptane, benzoic, dichloroacetic, undecanoic, and succinic acids [36] while P-BB12 has acetic, formic, and succinic acids [37], which can be considered as a safe candidate as an antimicrobial active liquid.

Changes in the psychrophilic counts of HMMC samples in the refrigerated condition are presented in Figure 1A. A significant statistical difference was observed between the treated samples and the control group over the course of the study, attributed to the antimicrobial properties of the compounds in the postbiotic solutions. The initial population of TPC was 5.5 log CFU/g in the control group, rising to 7.1 log CFU/g following 16 days of storage. Postbiotic solutions inhibited the psychrophile growth for at least 8 days, followed by a gradual increase in the TPC until the end of storage. The highest reduction (i.e., 1.5 log CFU/g) in TPC was reported for the P-BB-12 by day 16, while P-LA5 and the P-A5 + P-BB12 combination displayed a similar inhibitory activity during storage with a maximum reduction of 1 log CFU/g in psychrophiles.

The mesophile population increased progressively within each treatment during the storage period (Figure 1B). At the end of the storage period (day 16), the cheeses treated with P-BB12, P-LA5, and P-LA5 + P-BB12 had a lower TMC than the control cheese. With respect to the upper limit of acceptability recommended for TMC in fresh cheese (e.g., 7 log CFU/g) [38], it could be observed that the control samples exceeded the limit after 4 days of storage (7.4 log CFU/g), while cheeses with postbiotic solutions attained the limit on day 12. The results indicate that all postbiotic solutions decreased the mesophile growth compared to the control samples. The prolonged shelf life could be attributed to the antimicrobial effects of the bioactive compounds of postbiotics, especially antimicrobial peptides, which have been reported to have favorable antimicrobial activity on Gram-positive and Gram-negative bacteria [27,39]. In a similar work, four days extension in HMMC was reported after using a sodium alginate coating with 3% potassium sorbate [22].

However, LAB counts in the control samples showed an increasing trend throughout the storage period, whereas in cheese with postbiotics, the LAB increased only between days 8 and 16 (Figure 1C). Compared to the control sample, the P-LA5, P-BB12, and P-LA5 + P-BB12 inhibited LAB by approximately 1 to 1.3 log CFU/g (Figure 1C). This reduction is associated with the organic acid content of the postbiotics used in this study, which may inhibit the LAB population. A similar reduction in LAB following postbiotic application has already been reported [15,40]. P-BB12 and P-LA5 + P-BB12 exhibited the highest inhibiting effect on LAB. A reduction in LAB growth would control the pH of the HMMC samples. Zappia et al. [26] reported the same behavior after using the bergamot juice concentrate in HMMC preserving liquid. It should be noted that in some cases, the inhibition attributable to a single postbiotic was greater than the combined application. Actually, when two postbiotics were combined, the level of all ingredients in each postbiotic was reduced due to the dilution. In addition, it seems that certain compounds of postbiotics have neutral or antagonistic activities after mixing two postbiotics. This requires further investigation to clarify this fact through conducting minimum inhibitory concentration (MIC) and fractional inhibitory concentration (FIC) tests.

Yeasts and molds are among the major microorganisms involved in HMMC spoilage [41]. Our findings clearly demonstrate the antifungal activity of P-LA5, P-BB12, and P-LA5 + P-BB12 in HMMC (Figure 1D). Compared to other microbial groups, a very noticeable reduction (~3 log CFU/g on day 4) in YMC was observed in cheeses with P-LA5, P-BB12, and P-LA5 + P-BB12 (Figure 1D), revealing the fungistatic and fungicide properties of postbiotic solutions. This feature could be ascribed to the presence of organic acids in the postbiotics of the investigated probiotics [28]. All of the microbiological analyses proved the positive effects of the P-LA5, P-BB12, +P-BB12 solutions to increase the shelf life of HMMC and triple the shelf life in relation to samples without postbiotics. 

### 3.2. pH Changes

**Figure 2:**
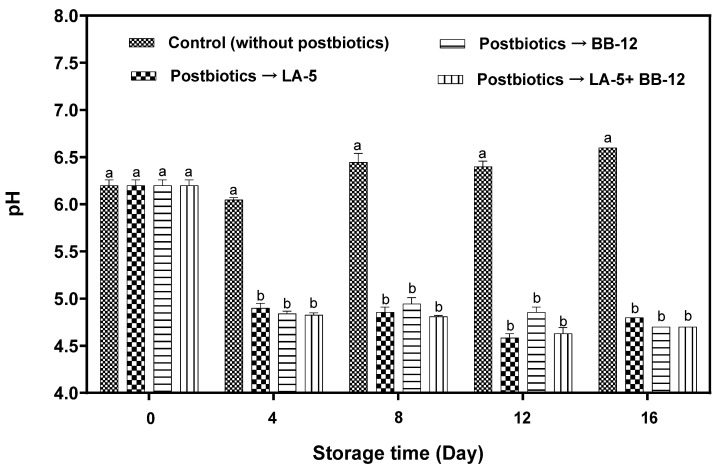
pH changes in mozzarella cheeses preserved in postbiotic solutions from *L. acidophilus* LA-5 (P-LA-5), *B. animalis* BB-12 (P-BB-12), and their combination (P-LA-5 + BB-12) during storage at 7 °C. Different lowercase letters indicate a significant (*p* < 0.05) difference between treatments on each storage day.

Figure 2 plots the changes in the pH of the cheeses stored in either water (control) or postbiotic solutions P-LA5, P-BB12, or P-LA5 + P212 BB12 over 16 days of storage at 7 °C. The initial pH of all cheese samples was 6.2 and after postbiotic use, a significant (*p* < 0.05) decrease in pH values was observed. This was associated with the presence of organic acids in the postbiotics absorbed by the cheese. Mass exchange is a possible reason for this modification. 

The lower pH of the cheese in the postbiotic samples was maintained throughout the storage period (*p* < 0.05), while a slight decrease in the pH of the control sample on the fourth day can be attributed to the activity of non-starter lactic acid bacteria [42]. In addition, an increase in the pH of control samples during the eighth, twelfth, and sixteen days of storage may be attributed to the pH of the preservative liquid and the mass exchange between HMMC and liquid (i.e., releases of citric acid from HMMC) [43]. The almost constant pH behavior of cheese in postbiotics after day 4 of storage could be associated with their protective activity against cheese decomposition (i.e., the high consistency of treated samples) and lower microbial growth compared to the control group. Earlier, a similar consistent pH for HMMC after applying active coatings with potassium sorbate, sodium benzoate, calcium lactate, and calcium ascorbate was reported [22].

### 3.3. Color Measurement

**Figure 3:**
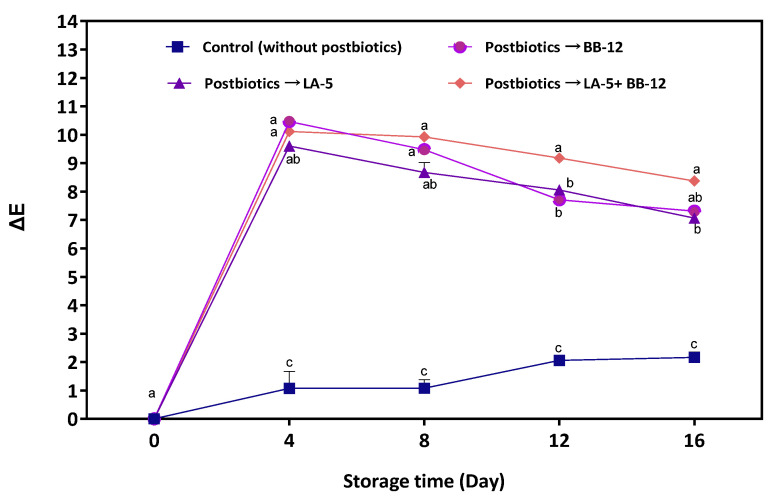
The color difference in cheese samples preserved in postbiotic solutions from *L. acidophilus* LA-5 (P-LA-5), *B. animalis* BB-12 (P-BB-12), and their combination during 16 days of storage. Different lowercase letters indicate a significant (*p* < 0.05) difference between treatments on each storage day.

∆E and L*, a*, b* of cheese with P-LA5, P-BB12, P-LA5 + P-BB12, and the control group are presented in Figure 3 and Table 1, respectively. According to the ΔE calculation, a significant (*p* < 0.05) color difference can easily be determined after postbiotic application (Figure 3). Meanwhile, the treatments and control samples were distinct with respect to the lightness of the colors (expressed as the L* value) and cheeses with postbiotics exhibited a decrease in the L* parameter and an increase in the a* parameter [red (+) to green (−)] and b* parameter [yellow (+) blue (−)].

The color shift in cheese samples may be related to the migration of postbiotic solutions to the surfaces of the cheese samples. The color of the postbiotics is linked to the whey solution used to prepare the postbiotics [44]. A color change in food samples after applying a postbiotic liquid is a common phenomenon [6], but it should be noted that these color changes are fairly minor compared to the use of postbiotics prepared in common laboratory culture media (i.e., MRS) instead of a whey solution. Similar color changes in cheese [17] and fresh beef [45] were reported following postbiotic application. However, the continuous color change in the control sample during storage was due to the proteolytic and lipolytic activities of psychrophiles [42].

### 3.4. Sensory Analysis

**Figure 4:**
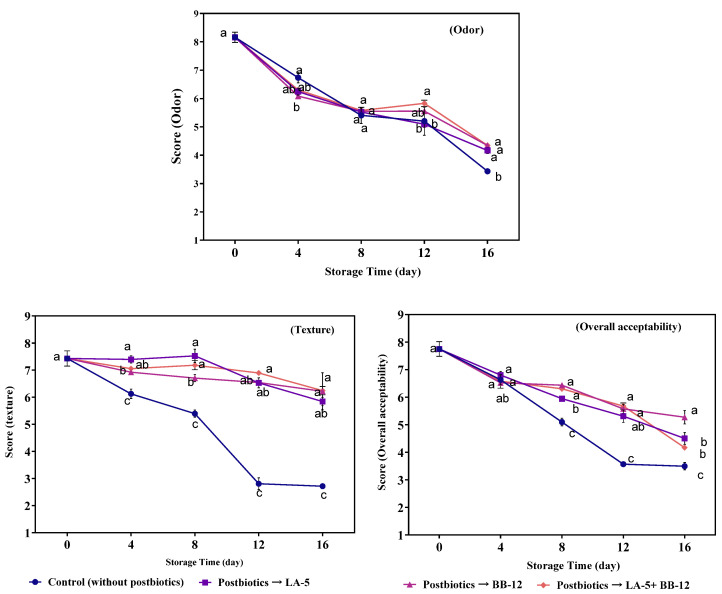
Sensory scores (odor, texture, and overall acceptability) in mozzarella cheese preserved in postbiotic solutions from *L. acidophilus* LA-5 (P-LA-5), *B. animalis* BB-12 (P-BB-12), and their combination during 16 days of storage. Different lowercase letters indicate a significant (*p* < 0.05) difference between treatments on each storage day.

The results of the sensory analyses (i.e., odor, texture, and overall acceptability) are presented in Figure 4. Based on the results of the sensory analyses, the control samples received the lowest scores in all indices with the exception of color by the assessors, while the highest scores were given for the P-LA5, P-BB12, and P-LA5 + P-BB12 cheeses over the storage period. This can be linked to the preservative activity of postbiotics by creating texture and a more pleasant smell. The desirable texture of the treated samples (Figure 4) may be due to the presence of exopolysaccharides in the postbiotic solutions [30]. In addition, the odor scores of all groups declined over time. The control samples exhibited a greater decline in odor scores compared to the treated samples. It has been reported that during the storage of cheese in a liquid solution, certain off-flavor volatile compounds derived from the activity of psychrophiles such as *Pseudomonas* spp. are released to the solution and their content is reduced over time within food [46]. In general, the assessors found no statistically significant difference in the overall acceptability of cheeses stored in postbiotics. The findings of similar work on the use of calcium lactate solution in HMMC showed that samples stored in calcium lactate solution had better sensory characteristics than the control sample [21].

## 4. Conclusions

The exploitation of new low-cost and commercially available sources for postbiotic preparation is particularly important. Whey is produced regularly in cheese plants, lost in most plants, and is considered as a waste in the food industry, where it can serve as an available source for postbiotic production. In this work, UF cheese whey was used as an alternative source for the preparation of postbiotics. Then, the effect of UF cheese whey containing postbiotics from *L. acidophilus* LA-5 and *B. animalis* BB-12 on the shelf life of HMMC was investigated. The results showed that the postbiotic solutions, in combination and individual forms, are effective in improving the shelf life of HMMC. However, in some cases, the impact of a single postbiotic solution on the microbial groups was greater than the combined form. The main factor for increasing the shelf life of HMMC up to 8 days was associated with the performance of postbiotic solutions in reducing the mesophile and psychrophile population. P-LA5 and P-BB12 had the greatest antibacterial effect on mesophiles and psychrophiles, respectively. Inhibiting these bacterial groups has a significant effect on the maintenance of the sensory properties of HMMC during storage. The postbiotic solutions derived from probiotics in cheese whey can represent viable candidates as preserving liquids in HMMC. However, postbiotic preparation in other animal and plant-based sources (waste or byproducts) can also be the subject of further investigation. Importantly, the regulations and labeling of food containing postbiotics are required for the commercial use of postbiotics in food.

## Figures and Tables

**Table 1:** Color change in the mozzarella cheese preserved in postbiotic solutions from *L. acidophilus* LA-5 (P-LA-5), *B. animalis* BB-12 (P-BB-12), and their combination during storage at 7 °C. Different lowercase letters indicate a significant (*p <* 0.05) difference between treatments on each storage day.

Treatment	Day	L*	a*	b*
Control	0	94.50 ± 0.80 ^a^	−2.51 ± 0.34 ^a^	10.06 ± 1.40 ^a^
	4	95.26 ± 0.30 ^a^	−3.16 ± 0.37 ^a^	10.03 ± 0.97 ^a^
	8	95.06 ± 0.95 ^a^	−3.41 ± 0.51 ^a^	10.60 ± 1.47 ^a^
	12	94.86 ± 4.78 ^a^	−3.16 ± 2.21 ^a^	11.46 ± 1.22 ^a^
	16	94.76 ± 0.90 ^a^	−2.91 ± 0.50 ^a^	12.16 ± 0.95 ^a^
P-LA-5	0	94.50 ± 0.80 ^a^	−2.51 ± 0.34 ^a^	10.06 ± 1.40 ^a^
	4	91.36 ± 2.82 ^b^	−2.31 ± 0.26 ^b^	18.76 ± 0.50 ^b^
	8	92.06 ± 2.61 ^b^	−1.21 ± 1.44 ^b^	18.50 ± 0.78 ^b^
	12	92.16 ± 3.15 ^b^	−1.62 ± 0.81 ^b^	18.66 ± 0.66 ^b^
	16	90.53 ± 1.73 ^b^	−0.93 ± 0.57 ^b^	17.46 ± 0.61 ^b^
P-BB-12	0	94.50 ± 0.80 ^a^	−2.51 ± 0.34 ^a^	10.06 ± 1.40 ^a^
	4	92.21 ± 2.56 ^b^	−2.41 ± 0.78 ^b^	20.03 ± 0.75 ^b^
	8	93.23 ± 1.05 ^b^	−1.73 ± 0.66 ^b^	19.16 ± 1.05 ^b^
	12	92.73 ± 0.43 ^b^	−1.81 ± 0.40 ^b^	18.53 ± 1.06 ^b^
	16	91.14 ± 2.17 ^b^	−1.43 ± 0.76 ^b^	18.33 ± 1.20 ^b^
P-LA-5 + BB-12	0	94.50 ± 0.80 ^a^	−2.51 ± 0.34 ^a^	10.06 ± 1.40 ^a^
	4	91.52 ± 2.38 ^b^	−2.16 ± 0.56 ^b^	19.40 ± 0.75 ^b^
	8	90.15 ± 0.78 ^b^	−0.96 ± 0.20 ^c^	18.90 ± 1.15 ^b^
	12	90.23 ± 1.20 ^c^	−1.36 ± 1.67 ^b^	18.93 ± 0.72 ^b^
	16	90.16 ± 2.45 ^b^	−0.91 ± 1.13 ^b^	18.70 ± 0.55 ^b^

## Data Availability

The datasets generated for this study are available on request to the corresponding author.
